# Strength and microstructure properties of solidified sewage sludge with two types of cement-based binders

**DOI:** 10.1038/s41598-020-77701-y

**Published:** 2020-11-27

**Authors:** Yijun Chen, Xingxing He, Shaohua Zhang, Xun Tan, Yong Wan

**Affiliations:** 1 State Key Laboratory of Geomechanics and Geotechnical Engineering, Institute of Rock and Soil Mechanics, Chinese Academy of Sciences, Wuhan, 430071 Hubei China; 2 University of Chinese Academy of Sciences, Beijing, 100049 China; 3 IRSM-CAS/HK PolyU Joint Laboratory on Solid Waste Science, Wuhan, 430071 China; 4 Hubei Key Laboratory of Contaminated Clay Science & Engineering, Wuhan, 430071 China; 5 Wuhan CAS-ITRI Solid Waste Resources Co., Ltd, Wuhan, 430014 China

**Keywords:** Civil engineering, Engineering, Pollution remediation

## Abstract

Solidification treatment with cementitious binder is an effective way to reduce environmental hazards of sewage sludge. Two cementitious binders, i.e., ordinary Portland cement (OPC) and sulfo-aluminate cement (SAC), were compared in this study to treat the sewage sludge. The strength of solidified sewage sludge (SSS) and changes in microscopic characteristics before and after treatment were analyzed through microscopic analysis methods. The effect of organic matter in sludge on the strength of SSS were also discussed. The results showed that the strength of SSS were lower than that of the solidified clay with no organic matter, and the filtrate extracted from the sludge can also weaken the cementation of the two cements significantly. The solidification effect of the OPC on the sludge was lower than that of the SAC evidently. The organic matter in the sewage sludge caused the surface of the soil particles to carry a large negative potential, which interfered with the hydration of the binder and reduced the amount of cementation skeleton formed by the binder hydration products. This resulted in a porous structure with low mechanical strength. The amount of early hydration product formed in the SAC-based solidified samples was higher than that of the OPC-based samples. This was favorable for filling the pores of the solidified samples and increasing their density. SAC had a better compatibility with soft soil containing high organic matter than OPC, and the which provides an effective alternative binder for dealing with sewage sludge.

## Introduction

Sewage sludge (SS) produced by water treatment plants tends to be characterized by high water content and high organic matter, showing the characteristics of easy flow and poor engineering strength^1–3^. In addition, some SS may also contain toxic and hazardous pollutants including microorganism, pathogen and heavy metals that may pollute the environment^4^. The safe disposal of SS has become an important issue affecting sustainable environmental management^5–9^. As an effective method, the solidification/stabilization treatment by mixing into a binder is widely used in the world to realize the resource utilization of SS^10–12^.

After adding the binder, the solidified waste can undergo a series of physicochemical reactions, which can stabilize the incorporated toxic substances and improve the mechanical property of SS simultaneously^12–16^. Then the physical and chemical properties of the SSS meet the requirements for disposal in a landfill or use in engineering applications, such as for bricks, subgrade and landfill cover^11,17–21^. Lim^15^ reported that the unconfined strength of the solidified sludge by fly ash and loess satisfied the criteria for construction materials, which was above 100 kPa. De Figueiredo et al.^19^ concluded that the sewage sludge added to soil and then solidified by lime can be used effectively as the base materials for roads and back filling. However, the selection of binder types and mixing ratios are the key factor to solidification effect.

The previous studies mainly used the OPC-based binder to treat soft soil^12,16^, but for the SS, the solidified effect is often poor due to the influence of organic matter^22^. That’s because the organic matter may interfere with the hydration and cementation process of binder, which can affect the overall solidification and subsequent disposal^23–27^. Since both the solid and pore fluid of SS are rich in organic matter, including protein, fat, and humus^28^, the influence mechanism of them on the solidification of SS is very complicated and is still under research. The sulfo-aluminate cement (SAC), as a common type of cement, has the characteristics of fast hardening, high strength and stable performance, and is widely used in some special projects^29^. Studies have shown that SAC can stabilize toxic and harmful substances well^30,31^. However, the effectiveness and mechanism of SAC treating SS with high organic matter are not well understood. Moreover, the components of SS, including clay, organic matter, and water, all may be the important factors influencing the engineering properties of SSS, few studies have discussed the effect of these components of sludge on the treatment, which will be considered and further researched in this study.

**Figure 1:**
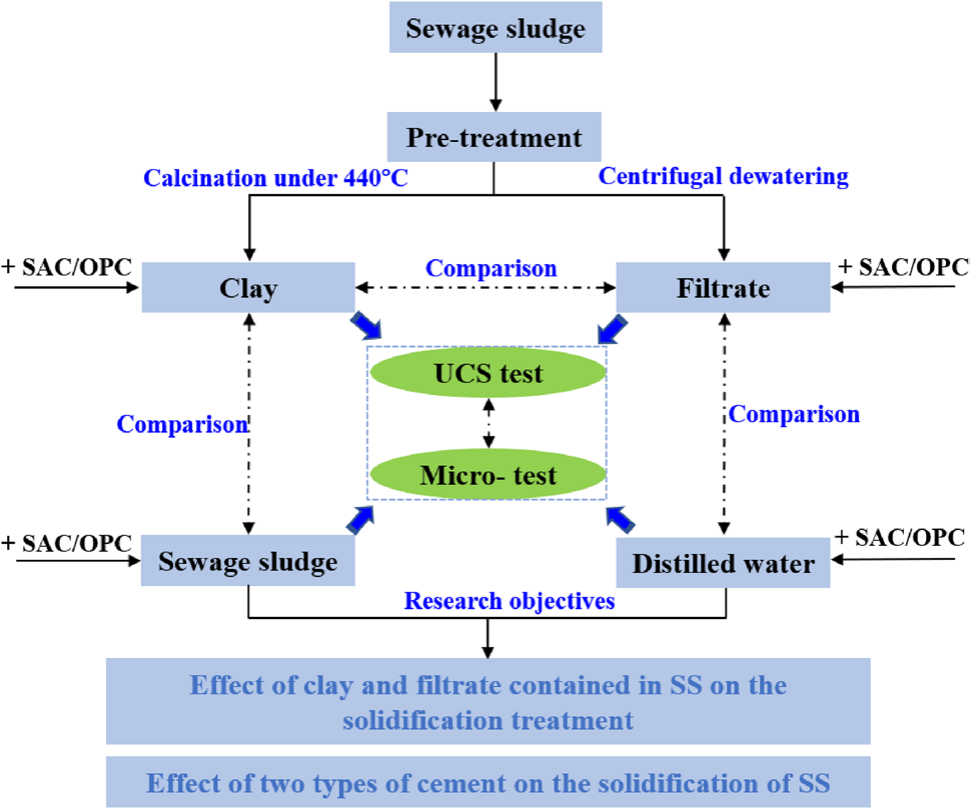
The flow chart of this study.

In this work, the solidification effect for SS with two types of cement-based binders (OPC and SAC) were compared. To study the effect of organic matter in sludge solids and in pore fluid on its solidification treatment, respectively, clay slurry obtained from SS, raw SS, filtrate of SS, and distilled water were used as the substrates for different solidification treatments. The unconfined compression strength of solidified samples by time were investigated. To reveal the solidification mechanism of SS with different organic matter content and different types of cement, a number of microscopic tests, including the zeta potential, X-ray diffraction (XRD), mercury intrusion porosimetry (MIP) and scanning electron microscopy analysis (SEM) were performed on the solidified samples. The flow chart of this study is shown in Fig. 1.

## Materials and methods

### Materials

**Figure 2:**
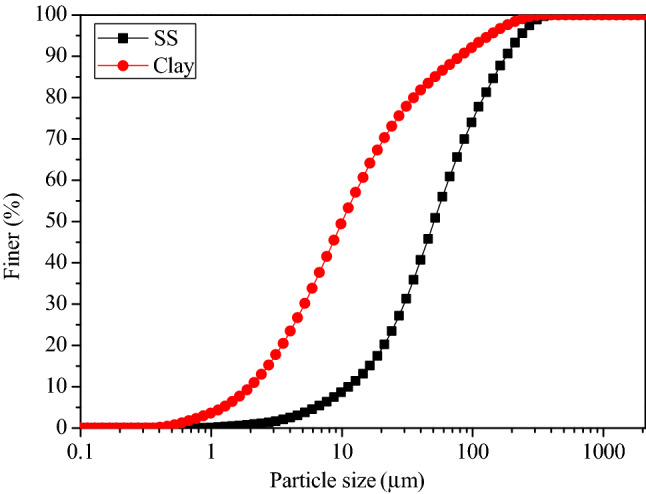
Particle size distribution of sewage sludge and clay.

**Figure 3:**
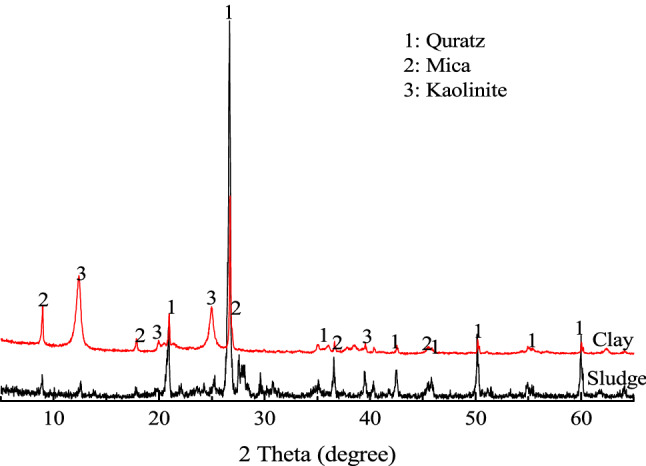
XRD patterns of the clay and sewage sludge.

The binders used in this study were type 42.5 OPC produced by Huaxin Cement Co., Ltd. and type 42.5 SAC produced by Dengdian Cement Group Co., Ltd. SS samples with an initial water content of 80% were taken from Hanxi Sewage Treatment Plant, Wuhan, China. After drying and breaking part of the sludge, put it in a furnace and burn it at 440 °C for 6 h, then the clay without organic matter can be obtained. Adding a certain amount of distilled water to the clay to prepare a clay slurry with a water content of 80% (as a mass ratio of water to the total clay slurry). The basic geotechnical properties of SS and clay are given in Table 1. The particle size distribution of raw SS and clay slurry were shown as Fig. 2, it can be found that the particles size of SS decrease obviously after removing organic matter. The XRD patterns of clay and SS are shown in Fig. 3. The filtrate (F) used in this study was obtained from the raw SS by a centrifuge method with velocities of 6000 rpm, and the total organic carbon (TOC) of filtrate was 1186 mg/L detected by a Multi N/C 2100 TOC analyzer (Analytik Jena, DE). As listed in Table 2, the elemental compositions of SS and the two types of cement are detected by X-ray fluorescence test.

**Table 1:** Index properties of sewage sludge and clay.

Physical index	Sewage sludge	Clay	Test standard
Nature water content, %	80.1	–	ASTM (2007)^32^
Specific gravity	2.55	2.67	ASTM (2014)^33^
Liquid limit (*ω*_*L*_), %	281.3	82.4	GB/T (2019)^34^
Plastic limit (*ω*_*P*_), %	78.2	46.3	GB/T (2019)^34^
Plastic index (*IP*)	203.1	36.1	
Clay fraction, %	1.06	10.26	
Silt fraction, %	63.49	78.89	
Sand fraction, %	35.45	10.85	
Diameter at 60%, μm	64.61	13.94	
Organic content (ignition loss), %	41.58	0	ASTM (2007)^32^
pH value	7.53	7.85	ASTM (2013)^35^
Unified soil classification system	CH	CH	ASTM (2017)^36^

**Table 2:** Elemental composition of raw materials.

Element	Sludge (wt%)	OPC (wt%)	SAC (wt%)
Mg	3.20	8.16	2.11
Al	14.36	5.03	14.34
Si	39.35	18.72	6.91
P	4.85	N.D	0.90
S	7.17	2.35	9.96
K	3.54	0.96	1.12
Ca	10.80	60.86	61.74
Ti	1.09	0.26	0.73
Cr	N.D	N.D	N.D
Mn	0.28	0.41	N.D
Fe	13.96	3.26	2.21
Cu	0.18	N.D	N.D
Zn	1.245	N.D	N.D

*N.D.* Not detected.

### Methods

#### Unconfined compressive strength

The solidified samples containing cement of OPC and SAC were numbered as OPC-X and SAC-X, respectively, as denoted in Table 3. All samples were composed of binder and water at a mass ratio of 1:1. The mixtures were stirred slowly by hand for 2 min, and then quickly in a cement mortar mixer for 5 min. The slurry mixture was filled to six forming molds (φ50 × 100 mm), and then cured in a curing chamber with temperature of 20 °C and 90% relative humidity for 24 h before demolding. The demolded samples were then further cured for some days and then the unconfined compressive strength was determined in accordance the standard ASTM (2016)^37^.

**Table 3:** Mix proportions of samples by weight.

Samples	Ordinary Portland cement	Sulfo-aluminate cement	Distilled water	Clay	Sludge	Filtrate of sludge
OPC-W	1	0	1	0	0	0
OPC-C	0.8^a^	0	0	1	0	0
OPC-S	0.8^a^	0	0	0	1	0
OPC-F	1	0	0	0	0	1
SAC-W	0	1	1	0	0	0
SAC-C	0	0.8^a^	0	1	0	0
SAC-S	0	0.8^a^	0	0	1	0
SAC-F	0	1	0	0	0	1

^a^This mass ratio ensures the water–cement ratio of these samples is 1:1.

#### Microstructure

To investigate the effect of the two binders on the surface potential of the sludge and common clay particles, the zeta potentials of dilute suspensions were measured, which was mixed and ultrasonicated for 5 min, and then standing for 5 min (the solutions had a water–solids ratio of 500:1 by weight) by means of a laser, Malvin NanoZS90, made in UK with 15 V loading. In the test process, the number of measurements was set to 10, and samples were tested at 20 ℃. To investigate the solidification mechanism of the cement-based binders, some small solidified blocks (20 mm × 20 mm × 20 mm) were also made for the MIP and SEM tests.

The test specimens were cured for 3, 7, 14, and 28 days and then crushed to determine the solidification reaction. The hydration of solidified samples was stopped using absolute ethanol, and the samples were dried under vacuum (less than 85 kPa) at – 40 ℃, then ground and screened. Pellets of less than 5 mm in diameter were selected for the MIP and SEM tests. MIP was performed using a PoreMaster 60GT (Quantachrome Instruments) by applying pressures from 0 to 28,850 psi (199 MPa). The instrument was capable of measuring pore size diameters down to 7 nm. The SEM tests were conducted using a JSM-6510 (Japan Electronics Company). Powders with sizes of less than 45 μm were used for XRD analysis with a scan range of 5°–70° and a scan rate of 10°/min. The XRD analyses were performed using a diffractometer, Smart Lab 3 kW, in a θ–θ configuration with Cu Kα radiation (λ = 1.5406 nm).

## Results and discussion

### Unconfined compressive strength

**Figure 4:**
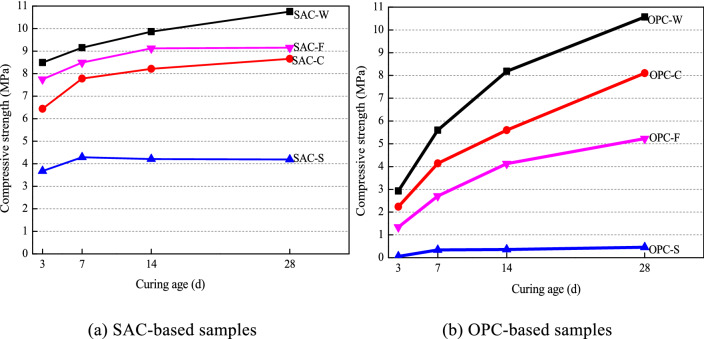
Compressive strength of the solidified cement-based samples at different curing ages.

Figure 4 shows the unconfined compressive strength of SAC-based and OPC-based solidified samples cured for 3, 7, 14, and 28 days. The compressive strength of each solidified sample generally increases with the curing time, and the strength varies for different types of soil. Compared with the blank samples (prepared with binder and distilled water), the raw SS has the most obvious weakening effect on the strength of solidified body than the clay and filtrate. By comparing the strength of SAC-S with OPC-S and SAC-F with OPC-F, respectively, it can be found that SAC is significantly higher than OPC for solidifying organic-rich substrates. The strength increases of SAC-based solidified samples mainly occurred in the first 7 days of curing, and for SAC-S samples, the strength at 7 days of curing has already reached its maximum value. But for the OPC-based solidified samples other than OPC-S, the strength has been increasing during the first 28 days of curing. At 7 days of curing, the strength of solidified SS with SAC was about 15 times that of SS solidified with OPC, and the solidified filtrate with SAC was about 3 times stronger than OPC.

Due to the large amount of organic matter contained in the filtrate, the strength of both OPC-F and SAC-F samples was significantly lower than that of the blank sample. Removing the organic matter from SS, the strength of the solidified clay by both types of cement would be greatly increased. Therefore, we can conclude that the organic matter in both the solids and filtrate of SS will reduce the solidification effect. After 28 days of curing, the strength of SAC-C is about 2 times that of SAC-S, and the strength of OPC-C can reach 20 times that of OPC-S. The strength of OPC-C and SAC-C samples was approximately the same at 28 days of curing, about 8.5 MPa. Thus, it can be easily obtained that SAC can effectively overcome the weakening effect of the organic matter and improve the strength of solidified SS, which may be related to the rapid cementation property of SAC.

### Zeta (ζ) potential analysis

According to the principles of colloid and interface science, the zeta potential of a colloid can reverse its overall stability. When the absolute value of the zeta potential (ζ) is small, the colloid is less stable and can condense quickly^38^. Therefore, ζ is an important factor reflects the binder reaction, this reaction in turn determines the early and late strength of the solidified samples. When suspensions in only water are considered, differences in the sign and magnitude of ζ are directly linked to the electrostatic charges existing on the surface of the SS particles.

Table 4 lists the ζ potential of the sludge, binders, and their solidified mixtures. The ζ potentials of clay, SS, SAC, and OPC are all negative. The ζ potential of SS is the smallest (− 16.2 mV) followed by the clay (− 11.8 mV). The ζ potential of SAC is − 4.4 mV, while that of OPC is − 0.2 mV. Adding cement to the clay, SS, and filtrate, the ζ potential of mixtures will reduce. The ζ potential of filtrate with cement binders is between those of SS and clay. This indicates that the electrical charge of the soil changes with the addition of cement binders, and organic matter has a greater influence on the electrostatic charges of solidified soils.

**Table 4:** Zeta potentials of raw materials before and after mixing with cementitious binder.

Composition	Clay	SS	SAC	OPC	SAC-C	SAC-S	SAC-F	OPC-C	OPC-S	OPC-F
ζ potential (mV)	− 11.8	− 16.2	− 4.4	− 0.2	− 7.8	− 10.5	− 6.3	− 7.1	− 11.4	− 6.8

### XRD analysis

**Figure 5:**
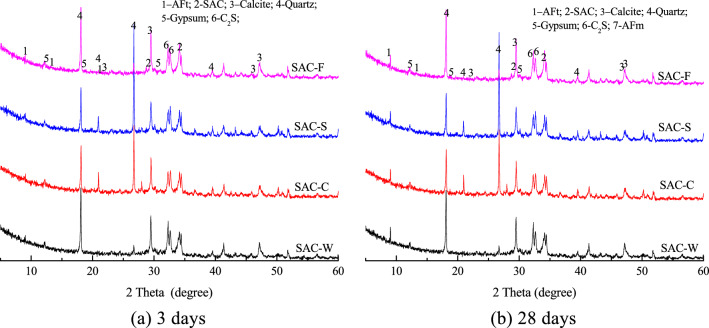
XRD patterns of SAC-based solidified specimens.

Figure 5 shows the XRD patterns for the clay, SS, and filtrate solidified with SAC and cured for 3 and 28 days. The crystalline phases in each sample are similar. Newly formed crystals of ettringite (AFt) are present in each solidified sample at 3 and 28 days, as well as incompletely reacted SAC clinker, gypsum, and calcium carbonate from the binders. Compared to the blank sample, large amounts of quartz and clay minerals, which were introduced from the soil, are present in the solidified clay, SS, and filtrate samples.

**Figure 6:**
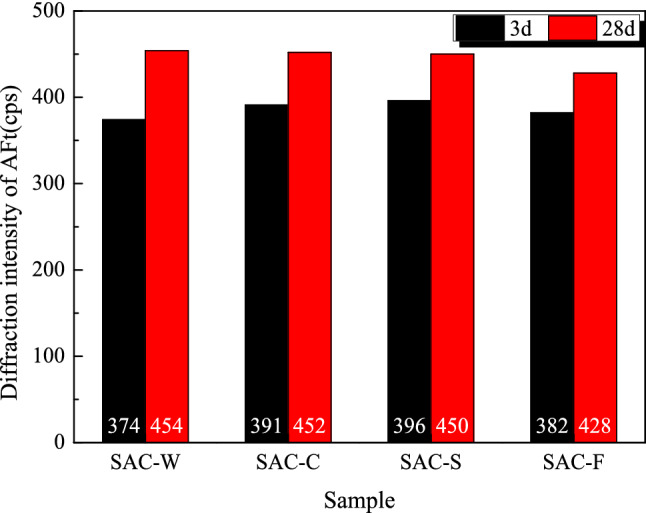
Diffraction intensity of the first diffraction peak of AFt for SAC-based solidified samples with different compositions at curing ages of 3 and 28 days.

Figure 6 shows the diffraction intensity of the first diffraction peak of ettringite in different samples cured for 3 and 28 days. The diffraction intensity of AFt in each sample is enhanced as the curing time increases (Fig. 3). At 3 days, the diffraction intensity of Aft differs for samples with different compositions. The AFt content the samples can be ranked as follows: SAC-S > SAC-C > SAC-F > SAC-W. The content of AFt in the solidified sample is greater than that in the blank sample, indicating that both clay and sludge promoted early reaction with the binder. At 28 days, the AFt content in the three types of solidified bodies is in the following order: SAC-W > SAC-C > SAC-S > SAC-F. Compared to the blank samples, the proportion of binder added in the three solidified samples is 80%. However, the AFt content of the three solidified samples is 94–99% that of the blank sample. These data indicate that the soil particles in the sludge promoted and participated in the reaction with the SAC binder.

**Figure 7:**
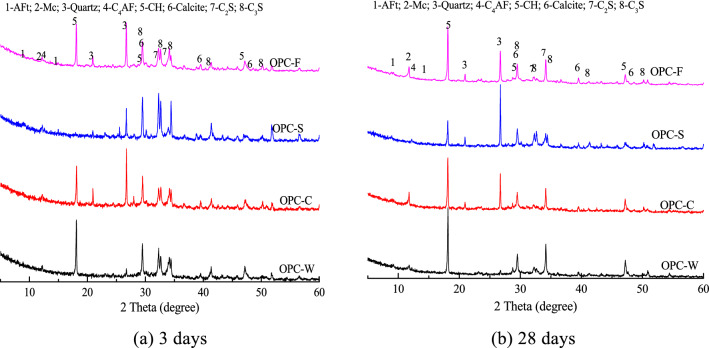
XRD patterns of OPC-based solidified samples.

Figure 7 shows the XRD patterns for clay and SS samples solidified with OPC and cured for 3 and 28 days. The phases present in the three solidified samples are similar. Newly formed ettringite, portlandite (CH), and mono-calcium aluminate crystals (Mc) are observed at 3 and 28 days. Moreover, clinkers such as C_4_AF, C_2_S, C_3_S, and calcium carbonate, which have not fully reacted with the soil, are also present. Substantial amounts of quartz are introduced from the soil in all three samples.

**Figure 8:**
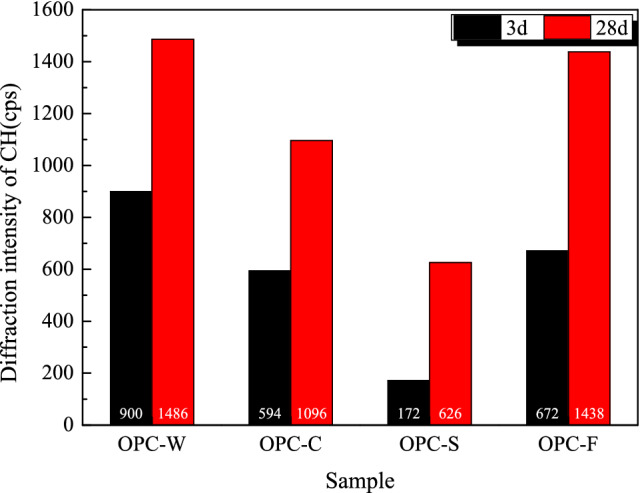
Diffraction intensity of the first diffraction peak of CH for OPC-based solidified samples with different compositions at curing ages of 3 and 28 days.

Figure 8 shows the diffraction intensity of the first diffraction peak of portlandite (CH) at 3 and 28 days in OPC-based samples with different compositions. The diffraction intensities of CH in the three solidified samples increase with increasing curing time. At 3 days, the diffraction intensity of CH differs slightly in the solidified samples. The diffraction intensity of CH in the blank sample is 900, which exceeds that of the other samples. Compared to the blank sample, the proportion of added binder in the three solidified samples is 80%. The diffraction intensity of CH in the solidified sludge is the lowest (172) and is less than 20% that of the blank. The CH contents of the OPC-F and OPC-C solidified samples are 75% and 66% that of the blank, respectively, indicating that clay and filtrate both inhibited the early reaction of the binder. At 28 days, the order of the CH content in the samples has changed to OPC-W > OPC-F > OPC-C > OPC-S. The CH content in the three solidified samples is lower than that in the blank sample; the CH content in the solidified sludge is only 42% that of the blank sample. These data indicate that the sludge with organic and inorganic particles inhibited the reaction of the binder.

### SEM analysis

**Figure 9:**
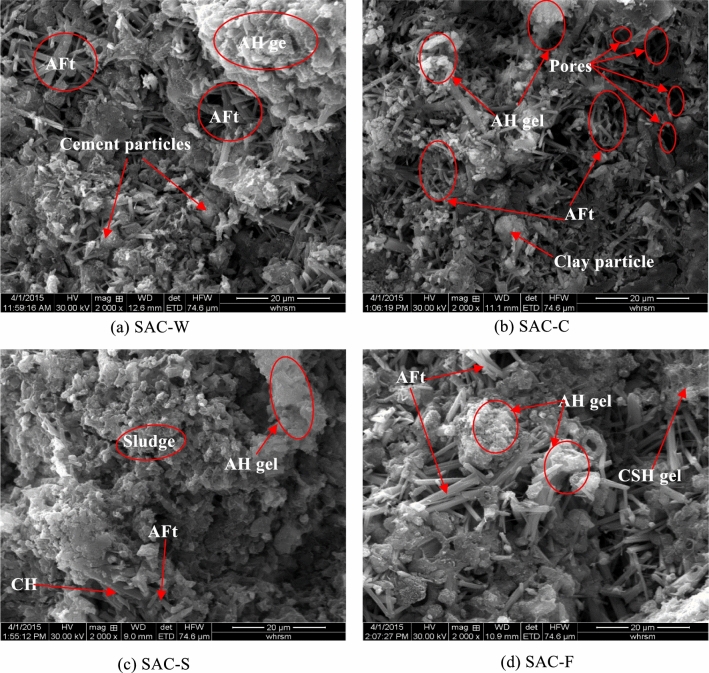
SEM images of SAC-based solidified samples cured for 28 days.

SEM images of the SAC-based solidified samples at 28 days of curing are shown in Fig. 9. A large amount of acicular and rod-shaped ettringite (AFt) are observed in these samples, as well as some aluminum hydroxide gel (AH)^39,40^. Hydration products such as AFt and AH are interwoven with incompletely reacted cement particles to form a dense structure in the blank sample, as shown in Fig. 9a. Figure 9b shows that in sample SAC-C, the newly formed acicular AFt and AH gel encapsulate the clay particles, forming a distinct interface transition zone. The ettringite and gel do not completely encapsulate the clay particles, and thus the sample contains a small number of pores. Compared to the sample in Fig. 9a, the SAC-C sample has a reduced density. As shown in Fig. 9c, small amount of ettringite can be observed in the SAC-S sample. This indicates that the hydration reaction of SAC can be significantly weakened by the sludge, however, a large number of AH gels can still be found in the SAC-S sample, which can slightly improve the strength of SS. Figure 9d shows that a large number of larger ettringite can be formed in SAC-F samples. The clay particles and organic particles contained in the filtrate are small and fill the voids formed from the cementation of binder particles. Moreover, the ettringite and alumina gel can further fill these voids, generating a sample containing a small number of micropores.

**Figure 10:**
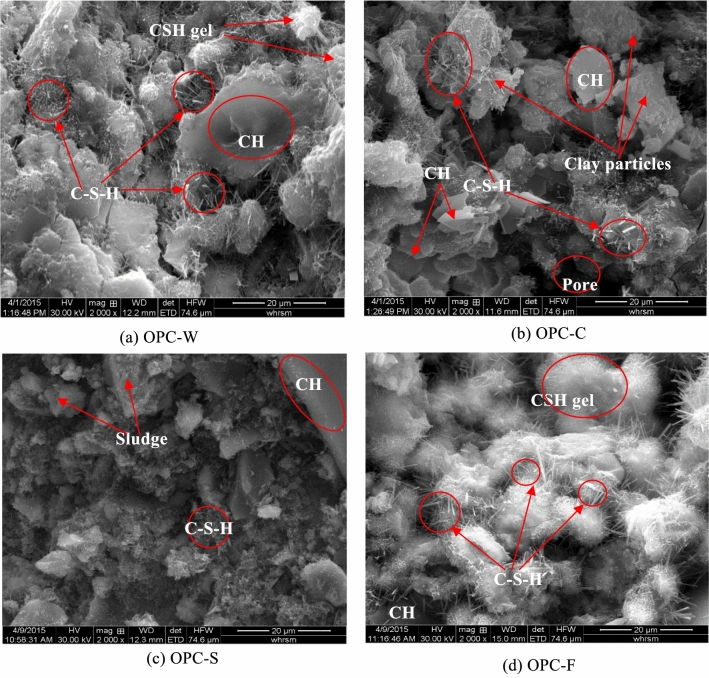
SEM images of OPC-based solidified samples cured for 28 days.

Figure 10 shows the SEM images of the OPC-based solidified samples at 28 days of curing. A large amount of fibrous calcium silicate gel (C–S–H) hydration products and calcium hydroxide (CH) can be identified in the blank sample, as shown in Fig. 10a. These C–S–H products were interlaced with a large amount of incompletely reacted cement particles to form a dense structure. Figure 10b shows that a large number of C–S–H products were attached to the surface of the clay particles. In addition, some early C–S–H gel also formed and enveloped the clay particles, showing a clear interface transition zone. However, the C–S–H gel cannot completely binder the clay particles, thus producing some sporadic pores in the sample. Compared to Fig. 10a,b, the amount of hydration product of C–S–H formed in OPC-S sample is limited and the size of these products are very small (Fig. 10c). A small amount of hydration products cannot effectively bind the sludge particles, resulting in low strength of solidified SS. As Fig. 10d shown, a large amount of fibrous C–S–H products were also generated in the OPC-F samples, however, the size of these products is small and many are in the form of early gel. This indicates that the organic matter contained in the filtrate of SS can also restrain the hydration of OPC.

### MIP analysis

Table 5 presents the total pore volume of each solidified sample at 28 days. The total pore volume of the SAC-W sample is between that of the SAC-F and SAC-C samples. The total pore volume of the SAC-S is the smallest of the SAC-based samples. The total pore volume of the OPC-W is slightly larger than that of OPC-F, but less than that of the OPC-S and OPC-C samples. The OPC-S has the largest total pore volume of the OPC-based samples. A comparison of the SAC- and OPC-based solidified samples shows that the total pore volumes of the SAC-based solidified samples are larger than that of the OPC-based samples, which is consistent with the SEM results. However, the total pore volume of OPC-F is smaller than that of SAC-F.

**Table 5:** Total pore volumes of cementitious solidified samples after curing for 28 days.

Sample	Total pore volume (ml g^−1^)	Sample	Total pore volume (ml g^−1^)
SAC-W	0.44	OPC-W	0.37
SAC-C	0.41	OPC-C	0.46
SAC-S	0.40	OPC-S	0.50
SAC-F	0.46	OPC-F	0.35

**Figure 11:**
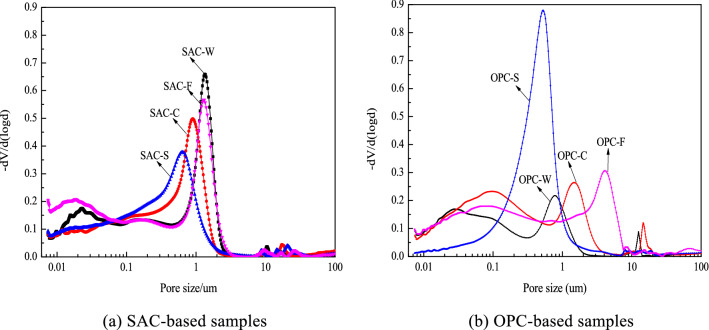
Pore size distribution of SAC- and OPC-based solidified samples at 28 days of curing.

Figure 11 shows the pore size distribution of the SAC- and OPC-based solidified samples at 28 days of curing. Compared to the SAC-W sample, the other SAC-based solidified samples have a wider pore size distribution and smaller pore size (Fig. 11a). The quantity of small pores increases and the most frequent pore size decreases. According to the SEM images above, the AFt and alumina gel produced by the reaction of the binder altered the original physical structure of SS. The porosity of the sample decreases and the density increases, but the interface bonding is weak, which affects the mechanical strength of the solidified samples.

Figure 11b shows that the porosity of each OPC-solidified sample increases compared to that of the OPC-W sample. The pore size of OPC-S becomes finer, and the pore size distribution widens and skews toward smaller pores; however, the maximum aperture size decreases. The pores of OPC-C and OPC-F become coarser. Their pore size distribution widens, and the size of the largest possible aperture increases. The number of macropores increases in the OPC-S and OPC-C samples (Table 5). The total pore volume increases, and the mechanical strength of the solidified samples decreases.

### Solidification mechanism of SOM with cement-based binder

When the binder is added to SS, the strength of the solidified sample is essentially improved by a series of physical and chemical reactions of the binder, these reactions will form a skeleton in soil^26,41^ and transform the SS from a soft plastic state to a solidified state. With increasing curing time, the reaction progression of the binder increases, and the strength of the solidified mixture gradually increases until it becomes stable. The properties of the binder and the base material are the major factors affecting the mechanical strength of the solidified samples. Compared to the SAC samples, the strength of the OPC-based samples developed slowly during the early curing stages but became faster in later stages. The effect of the two cements on the solidification differed for the clay, SS, and filtrate. Generally, the strength of the SAC-based solidified samples was greater than that of the OPC-based samples. The strength of SAC-S at 28 days of curing was 4.10 MPa, which is much higher than that of the OPC-S (0.46 MPa). In the SAC-based solidified samples, the SEM images show that the generation of ettringite and alumina gel were almost unaffected by the base material (Fig. 9). However, the products of the OPC-based solidified samples were affected by changes in the base material.

In addition to the specific hydration properties of the binder, the significant differences between the effects of the two binders on the solidification of clay and SS were related to the properties of the base material (particle size, surface properties of particle, and mineral phase composition)^41,42^. In this study, the physicochemical properties of the clay and SS were similar, but the difference in the organic matter content between the two was significant, which led to considerably different solidification effects. Organic matter can affect the solidification because it has surface properties that are different from the inorganic binder. The difference was mainly seen in the solidification process for the binder with SS. Soil with a high organic matter content has a large amount of surface negative charge (Table 4), which enhances its stability and increases the distance between the binder particles and SS particles. Moreover, negative electrical charges are mutually exclusive, i.e., they lower the early reaction rate in solidified SS. Some organic matter was adsorbed onto the surface of the binder particles. This resulted in a negative initial zeta potential of the solidified mixture, and affected the reaction of the binder. The most direct manifestation of this is that the solidified sample had less reaction product (CH) than the other samples (Fig. 8), then forming a porous structure and reduced density.

Based on the results of the previous test, the TOC of the sludge filtrate is very high (1186 mg/L), which indicates that a large amount of organic matter in the sludge is directly dissolved in the pore solution. Considering the main components of sludge, it can be considered that the factors that contribute to weakening the strength on the solidified samples arise from three components: the clay particles (C), organic matter attached to the clay particles (SS), and sludge filtrate (F). By comparing the strength of the blank samples under the two types of binders, the degree of weakening attributed to these factors can be ordered for the SAC binder as SS > F > C. Meanwhile, for the OPC binder, it is SS > C > F. Therefore, it can be considered that the organic matter adsorb on clay is the most important factor in weakening the strength of solidified samples, and because the sludge filtrate contains some organic matter, it also has a certain weakening effect on the strength. Moreover, the weakening effect with the OPC binder is obviously stronger than that with the SAC binder.

## Conclusions

The main conclusions of this study are as follows:

1. The effects of SAC and OPC on the solidification of SS were significantly different. The strength of SSS solidified by SAC was far greater than that of the OPC-based samples. However, the strength of solidified clay (without organic matter) with SAC was similar to that of the OPC-based sample at 28 days of curing. Therefore, SAC is better for solidifying soft soil with high organic matter.
2. The organic matter contained in both filtrate and clay can weaken the solidification effect of sewage sludge. Organic matter changes the soil charge and the pore size of the solidified samples, which is an essential factor affecting the cementation effect of cement in SS.
3. The mechanical strength of the solidified SS is closely related to its microstructure, and the denser the skeleton structure of the solidified sample, the higher the corresponding strength will be. The total pore volume contained in the higher-strength solidified samples is smaller, but the most probable pore size has a tendency to increase.

## References

[1] Yang J (2017). Direct reuse of two deep-dewatered sludge cakes without a solidifying agent as landfill cover: Geotechnical properties and heavy metal leaching characteristics. RSC Adv..

[2] Diliunas J, Dundulis K, Gadeikis S, Jurevicius A, Kaminskas M (2010). Geotechnical and hydrochemical properties of sewage sludge. Bull. Eng. Geol. Environ..

[3] Tan X, Chen Y, Xue Q, Wan Y, Liu L (2020). Conditioning of resuspension excess sludge with chemical oxidation technology: The respective performance of filtration and expression stage in compression dewatering. Sep. Purif. Technol..

[4] Liu J, Chen J, Huang L (2015). Heavy metal removal from MSS fly ash by thermal and chlorination treatments. Sci. Rep..

[5] Feng LY, Luo JY, Chen YG (2015). Dilemma of sewage sludge treatment and disposal in China. Environ. Sci. Technol..

[6] Peccia J, Westerhoff P (2015). We should expect more out of our sewage sludge. Environ. Sci. Technol..

[7] Guo XF (2020). Heavy metals removal from sewage sludge with mixed chelators of N,N-bis(carboxymethyl) glutamic acid and citric acid. Environ. Technol..

[8] Jablonska-Trypuc A, Wydro U, Serra-Majem L, Butarewicz A, Wolejko E (2019). The comparison of selected types of municipal sewage sludge filtrates toxicity in different biological models: From bacterial strains to mammalian cells. Preliminary Study. Water.

[9] Chen X (2020). Sludge biochar as a green additive in cement-based composites: Mechanical properties and hydration kinetics. Constr. Build. Mater..

[10] Zhen GY (2011). Effects of calcined aluminum salts on the advanced dewatering and solidification/stabilization of sewage sludge. J. Environ. Sci. China.

[11] Li Y (2014). Reuse of dewatered sewage sludge conditioned with skeleton builders as landfill cover material. Int. J. Environ. Sci. Technol..

[12] Lin, C., Zhu, W. & Han, J. In *Contemporary Topics in Ground Modification, Problem Soils, and Geo-Support* 281–288 (2009).

[13] Wang D, Abriak NE, Zentar R (2013). Strength and deformation properties of Dunkirk marine sediments solidified with cement, lime and fly ash. Eng. Geol..

[14] Lin C, Zhu W, Han J (2013). Strength and leachability of solidified sewage sludge with different additives. J. Mater. Civ. Eng..

[15] Lim S, Jeon W, Lee J, Lee K, Kim N (2002). Engineering properties of water/wastewater-treatment sludge modified by hydrated lime, fly ash and loess. Water Res..

[16] Chen QY, Tyrer M, Hills CD, Yang XM, Carey P (2009). Immobilisation of heavy metal in cement-based solidification/stabilisation: A review. Waste Manag..

[17] Kalpokaitė-Dičkuvienė R (2018). Utilization of sewage sludge-biomass gasification residue in cement-based materials: Effect of pozzolant type. Environ. Technol..

[18] Jiang DL, Ni GW, Ma GY (2011). Reuse of municipal wastewater sludge for construction material. Adv. Mater. Res.-Switz..

[19] Lucena L, Juca JFT, Soares JB, Portela MG (2014). Potential uses of sewage sludge in highway construction. J. Mater. Civ. Eng..

[20] Taki K, Choudhary S, Gupta S, Kumar M (2020). Enhancement of geotechnical properties of municipal sewage sludge for sustainable utilization as engineering construction material. J. Clean. Prod..

[21] He X, Chen Y, Wan Y, Liu L, Xue Q (2020). Effect of curing stress on compression behavior of cement-treated dredged sediment. Int. J. Geomech..

[22] Alqedra M, Arafa M, Mattar M (2011). Influence of Lovv and high organic wastevvater sludge on physical and mechanical properties of concrete mixes. J. Environ. Sci. Technol..

[23] Chen H, Wang Q (2006). The behaviour of organic matter in the process of soft soil stabilization using cement. Bull. Eng. Geol. Environ..

[24] Tremblay H, Duchesne J, Locat J, Leroueil S (2002). Influence of the nature of organic compounds on fine soil stabilization with cement. Can. Geotech. J..

[25] Harvey OR, Harris JP, Herbert BE, Stiffler EA, Haney SP (2010). Natural organic matter and the formation of calcium-silicate-hydrates in lime-stabilized smectites: A thermal analysis study. Thermochim. Acta.

[26] Bobet A, Hwang JH, Johnston CT, Santagata M (2011). One-dimensional consolidation behavior of cement-treated organic soil. Can. Geotech. J..

[27] Kang G-O, Tsuchida T, Kim Y-S, Baek W-J (2017). Influence of humic acid on the strength behavior of cement-treated clay during various curing stages. J. Mater. Civ. Eng..

[28] Poykio R, Watkins G, Dahl O (2019). Characterisation of municipal sewage sludge as a soil improver and a fertilizer product. Ecol. Chem. Eng. S.

[29] Mobili A, Telesca A, Marroccoli M, Tittarelli F (2020). Calcium sulfoaluminate and alkali-activated fly ash cements as alternative to Portland cement: study on chemical, physical-mechanical, and durability properties of mortars with the same strength class. Constr. Build. Mater..

[30] Luz CA, Rocha JC, Cheriaf M, Pera J (2006). Use of sulfoaluminate cement and bottom ash in the solidification/stabilization of galvanic sludge. J. Hazard. Mater..

[31] Luz CA, Rocha JC, Cheriaf M, Pera J (2009). Valorization of galvanic sludge in sulfoaluminate cement. Constr. Build. Mater..

[32] ASTM. Standard test methods for moisture, ash, and organic matter of peat and other organic soils. *ASTM International, West Conshohocken* D2974-07 (2007).

[33] ASTM. Standard test methods for specific gravity of soil solids by water pycnometer. *ASTM International, West Conshohocken* D854-14 (2014).

[34] GB/T. Standard for Geotechnical Testing Method. *The Ministry of Water Resources of the People’s Republic of China, Beijing: China Planning Press.* GB/T50123 (2019).

[35] ASTM. Standard test method for pH of soils. *ASTM International, West Conshohocken, PA* D4972-13 (2013).

[36] ASTM. Standard Practice for Classification of Soils for Engineering Purposes (Unified Soil Classification System). *ASTM International, West Conshohocken, PA* D2487-17e1 (2017).

[37] ASTM. Standard Test Method for Unconfined Compressive Strength of Cohesive Soil. *ASTM International, West Conshohocken, PA* D2166M-16 (2016).

[38] García-García S, Jonsson M, Wold S (2006). Temperature effect on the stability of bentonite colloids in water. J. Colloid Interface Sci..

[39] Gastaldi D (2016). Hydration products in sulfoaluminate cements: Evaluation of amorphous phases by XRD/solid-state NMR. Cem. Concr. Res..

[40] Lin RS, Wang XY, Lee HS, Cho HK (2019). Hydration and microstructure of cement pastes with calcined Hwangtoh clay. Materials.

[41] Zhang R, Santoso A, Tan T, Phoon K (2013). Strength of high water-content marine clay stabilized by low amount of cement. J. Geotech. Geoenviron. Eng..

[42] Chrysochoou M, Grubb DG, Drengler KL, Malasavage NE (2010). Stabilized dredged material. III: Mineralogical perspective. J. Geotech. Geoenviron. Eng..

